# Cerebellar rTMS and PAS effectively induce cerebellar plasticity

**DOI:** 10.1038/s41598-021-82496-7

**Published:** 2021-02-04

**Authors:** Martje G. Pauly, Annika Steinmeier, Christina Bolte, Feline Hamami, Elinor Tzvi, Alexander Münchau, Tobias Bäumer, Anne Weissbach

**Affiliations:** 1 Institute of Systems Motor Science, University of Lübeck, Ratzeburger Allee 160, 23562 Lübeck, Germany; 2 Institute of Neurogenetics, University of Lübeck, Ratzeburger Allee 160, 23562 Lübeck, Germany; 3 Department of Neurology, University Hospital Schleswig Holstein, Ratzeburger Allee 160, 23538 Lübeck, Germany; 4 Department of Neurology, University of Leipzig, Liebigstraße 20, 04103 Leipzig, Germany

**Keywords:** Neurology, Motor cortex, Cerebellum, Premotor cortex

## Abstract

Non-invasive brain stimulation techniques including repetitive transcranial magnetic stimulation (rTMS), continuous theta-burst stimulation (cTBS), paired associative stimulation (PAS), and transcranial direct current stimulation (tDCS) have been applied over the cerebellum to induce plasticity and gain insights into the interaction of the cerebellum with neo-cortical structures including the motor cortex. We compared the effects of 1 Hz rTMS, cTBS, PAS and tDCS given over the cerebellum on motor cortical excitability and interactions between the cerebellum and dorsal premotor cortex / primary motor cortex in two within subject designs in healthy controls. In experiment 1, rTMS, cTBS, PAS, and tDCS were applied over the cerebellum in 20 healthy subjects. In experiment 2, rTMS and PAS were compared to sham conditions in another group of 20 healthy subjects. In experiment 1, PAS reduced cortical excitability determined by motor evoked potentials (MEP) amplitudes, whereas rTMS increased motor thresholds and facilitated dorsal premotor-motor and cerebellum-motor cortex interactions. TDCS and cTBS had no significant effects. In experiment 2, MEP amplitudes increased after rTMS and motor thresholds following PAS. Analysis of all participants who received rTMS and PAS showed that MEP amplitudes were reduced after PAS and increased following rTMS. rTMS also caused facilitation of dorsal premotor-motor cortex and cerebellum-motor cortex interactions. In summary, cerebellar 1 Hz rTMS and PAS can effectively induce plasticity in cerebello-(premotor)-motor pathways provided larger samples are studied.

## Introduction

The cerebellum is an important relay in motor networks and has a crucial role in movement execution and control, not only by modulating primary motor cortex (M1) output through cerebello-thalamo-cortical pathways^1^, but also via basal ganglia^2,3^ and brainstem connections^4^. In particular, actions can be modulated by the cerebellum through corrective signals to the brainstem to alter motor execution or via thalamo-cortical projections to modulate motor preparation, because the cerebellum balances motor intention with motor execution^4^. Thus, studies have shown that error-based adaptation tasks are cerebellar dependent^5^. Furthermore, a di-synaptic pathway between the cerebellum and the basal ganglia has shown to link cerebellar error-based motor learning to reinforcement motor learning mediated by the basal ganglia^6^. Another key player in the motor network is the (dorsal) premotor cortex (PMd) due to its major role in movement preparation and shaping, as well as the execution of externally guided movements^7^. Studies based on retrograde transneuronal virus tracing^8^, computational modeling^9^, and TMS^10^ suggest a connection between the cerebellum and the premotor cortex.

Cerebellar connectivity with cortical motor areas can be measured non-invasively using transcranial magnetic stimulation (TMS), for instance by pairing magnetic pulses applied over the cerebellum and these areas. Activation of the dentato-thalamo-cortical tract increases M1 excitability^11^. Purkinje cell activation in turn inhibits this pathway^11^. Therefore, cerebellar TMS, supposedly activating Purkinje cells^12,13^, is expected to cause a net inhibition of M1. This has indeed been documented. Conditioning TMS pulses applied to the cerebellum 5 to 6 ms prior to M1 stimulation, reduce motor evoked potential (MEP) amplitudes as a measure of cortico-spinal excitability^14^. This has been referred to as cerebellar brain inhibition (CBI) and has been studied both in healthy subjects^5,15–17^ and patients with neurological diseases^18–23^. CBI has also been shown to have an effect on intracortical excitability such as short-interval intracortical inhibition (SICI)^21^. Furthermore, single-pulse cerebellar TMS has been shown to reduce contralateral silent periods at interstimulus intervals (ISI) between 20 and 40 ms^24^. Whether activation of premotor cortical areas contributes to CBI has not been investigated as yet.

Since interactions between cerebellum, PMd, and M1 (Cerebello-PMd-M1) may underlie motor network plasticity processes such as motor sequence learning^25^ and visuomotor adaptation^26^, triple-pulse TMS seems to be a promising tool to gain further insights into cerebello-PMd-M1 connectivity. PMd-M1 interaction has been shown to depend on the trimming of pulses, the intensity of the PMd conditioning pulse and ISI between PMd and M1 pulses. MEP amplitude reductions were induced by PMd conditioning pulses with an intensity of 90% of active motor threshold (AMT) at ISIs of 4–6 ms^27^ in younger healthy controls, whereas higher intensities were needed in older subjects^28^. Whether cerebellar influence on M1 is mediated through potentially time-sensitive connections with PMd is unknown. How cerebellar excitability changes could contribute to PMd-M1 connectivity is also unclear.

Non-invasive brain stimulation (NIBS) such as repetitive TMS (rTMS) or transcranial direct current stimulation (tDCS) can be used to induce such longer-lasting excitability changes, i.e. plasticity, in different areas of the brain, including the cerebellum^29^. Only few cerebellar NIBS plasticity protocols have been tested and results of these studies were variable^30^. TDCS applied over the motor cortex is considered to affect cortical excitability through alterations of the resting membrane potential^30^ as a function of polarity with decreased excitability after cathodal and increased excitability after anodal tDCS^31^. Administered over the cerebellum, anodal tDCS has been shown to decrease the threshold for inducing CBI, which was interpreted as increased excitability of the cerebellar cortex, since M1 excitability was not affected. Cathodal stimulation resulted in opposite effects^32,33^. Studies in patients with ataxia showed clinical improvement after anodal tDCS^34,35^. The influence of cerebellar tDCS on CBI is equivocal with some studies showing decreased^15,36^ CBI and others, as pointed out above, reduced thresholds to induce CBI^33^ after anodal tDCS.

Conventional low-frequency rTMS, e.g. 1 Hz, has consistently been shown to reduce M1 excitability when applied to M1^37–41^. Application over the cerebellum however led to MEP facilitation, probably due to a transiently reduced excitability of Purkinje cells and an increased excitability of spinal alpha-motorneurons^42,43^. A continuous theta-burst stimulation (cTBS) protocol, in which rapid trains of TMS pulses are given at an interpulse interval of 50 Hz, has on the other hand been shown to decrease MEP amplitudes when applied over M1^44^, as well as over the cerebellum^16^. However, the effect of cTBS is subject to high inter-subject-variability^45–47^. Another plasticity inducing technique is paired associative stimulation (PAS). Originally, TMS stimulation over M1 was combined with electrical stimulation of the contralateral median nerve. This combination of somatosensory input and activation of M1 resulted in Hebbian plasticity and changes of corticospinal excitability depending on the ISI^48^. Using a PAS protocol where cerebellar TMS stimulation was coupled with M1 TMS pulses, MEP inhibition occurred when M1 TMS pulses were preceded by cerebellar pulses by 6 ms^17^.

A large number of studies using cerebellar NIBS with different protocols have shown variable effects on motor cortical excitability and intracortical interactions in healthy controls and patients^30,49^. Whereas reviews have contrasted different NIBS protocols across studies^30,49^, such comparisons have the disadvantage that NIBS protocols have been applied in different populations. The main aim of the present study was a direct comparisons of different NIBS techniques in the same group of participants. To the best of our knowledge, this is the first study directly comparing the effects of rTMS, cTBS, tDCS, PAS, and sham stimulation in the same group of probands. Here, we investigated the effect of the aforementioned cerebellar plasticity protocols on M1 excitability, as well as PMd-M1 and cerebello-PMd-M1 connectivity, in healthy controls, using multi-coil, paired-pulse TMS with the aim to determine the effectiveness of these measures and their potential usefulness as treatment tools in patients with neurological disorders.

## Methods

### Participants and study design

In experiment 1, we investigated 20 healthy righthanded subjects (13 female, mean age 27 ± 2 years standard error of mean), who did not report any neurological disorders or symptoms, by comparing four different cerebellar plasticity induction techniques that have been effective in other studies, i.e. 1 Hz rTMS^50^, PAS^17^, cTBS^51^ and tDCS^32^. The order of the four plasticity techniques was randomized and they were applied at least one week apart from each other. Pre and post plasticity induction, cortical excitability was probed by single-pulse TMS determining resting motor threshold (RMT), AMT and MEPs, as well as dual-pulse TMS protocols for charting left PMd-M1 and cerebello-M1 excitability. Moreover, a triple-pulse TMS paradigm was used to investigate cerebello-PMd-M1 interactions.

**Figure 1:**
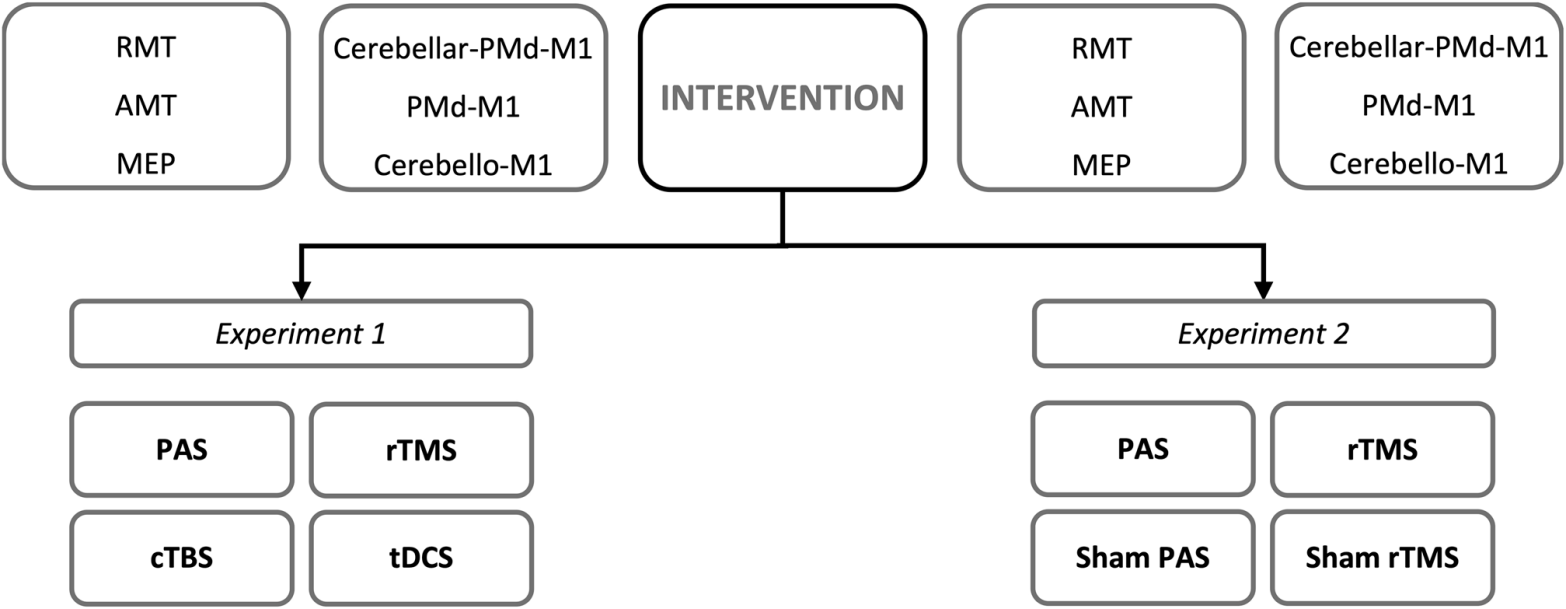
Study design. Probands were investigated in experiments 1 or 2 using four separate sessions with one of the four different plasticity intervention. Before and after the intervention the same single- and multi-pulse TMS paradigms were applied. *RMT* resting motor threshold, *AMT* active motor threshold, *MEP* motor evoked potential, *PMd* dorsal premotor cortex, *M1* primary motor cortex, *PAS* paired associated stimulation, *rTMS* repetitive transcranial magnetic stimulation, *cTBS* continuous theta-burst, *tDCS* transcranial direct current stimulation.

In experiment 2, we re-evaluated the efficacy of 1 Hz rTMS and PAS, which turned out to induce significant effects in experiment 1, complemented by sham stimulation as control conditions, i.e. rTMS sham and PAS sham. In experiment 2, another group of 20 healthy controls (13 female, mean age 27 ± 1.93 years standard error of mean) were investigated of whom two participants already took part in experiment 1. Pre and post plasticity measurements were identical in experiments 1 and 2 (Fig. 1).

### Experimental setup

The experimental setup was similar to our previous studies^52,53^. Electromyography was measured over the right first dorsal interosseus muscles (FDI) by Ag/Ag–Cl disc surface electrodes in a belly tendon montage. Electromyography signal was filtered and amplified by a D360 amplifier (Digitimer Limited, Welwyn Garden City, Hertfordshire, UK) and subsequently digitized and recorded by a laboratory interface (Micro 1401; Cambridge Electronics Design (CED), Cambridge, UK) and SIGNAL software (Cambridge Electronic Devices, Cambridge, UK).

### Neuronavigation

Neuronavigation was used to track the TMS coils and the subjects head to mark targets previously identified in the MRI on the scalp. The Brainsight neuronavigation system (Rogue Research, Montreal, Canada) was used in combination with the Polaris camera (Northern Digital, Ontario, Canada). Target regions for left PMd and M1 as well as for right cerebellum were identified anatomically by using an individual T1-weighted MRI for each subject. PMd was located in the gyrus anterior of the hand knob and lateral of the sulcus frontalis superior, in close proximity to the anatomical M1 (hand knob). The location of M1 was verified by identifying the “motor hot spot”, the location where a TMS pulse administered at a supra-threshold intensity produced continuously the highest MEP. Due to the close proximity of PMd and M1, the stimulation site of PMd had to be adjusted and moved anterior in some individuals, since placement of one coil on the previously identified PMd site and another coil close enough to the “motor hot spot” to generate a sufficient MEP was not feasible. The adjusted PMd location was recorded using the brain sight software.

**Figure 2:**
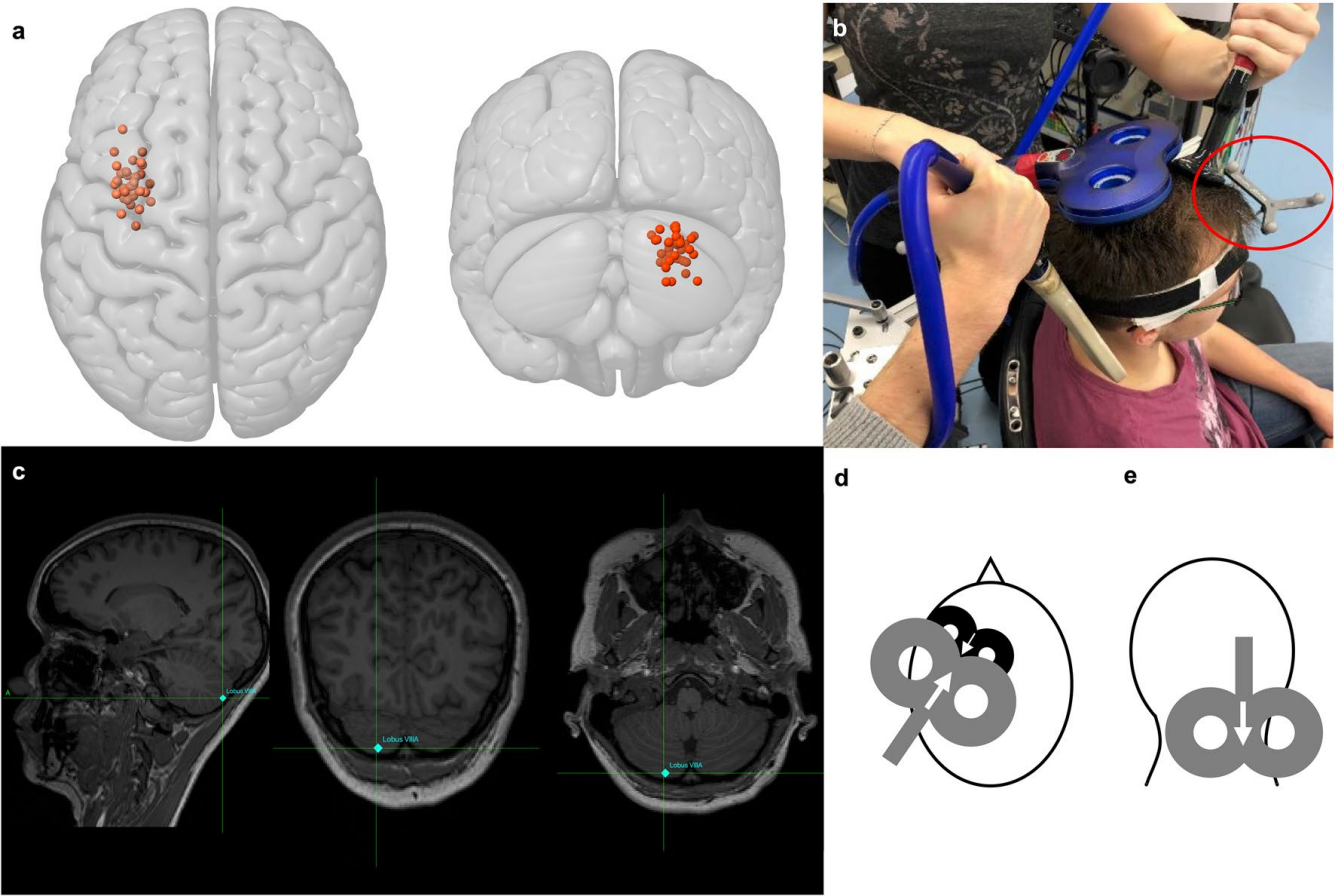
Coil positions and stimulation sites. (**a**) Individual stimulation sites for right lobule VIIIA (red) and dorsal premotor cortex (PMd) (orange) using the Montreal Neurological Institute and Hospital (MNI) stimulation coordinates in anatomical convention. (**b**) Neuronavigated triple-coil transcranial magnetic stimulation (TMS) experimental setup. The red circle marks the position of the tracer with three reflecting spheres on the subject’s head (staff member, that gave permission to publish the picture). TMS coils are located over PMd (small 25 mm black coil) and cerebellum stimulation site (grey figure of eight coil). Blue 70 mm coil is located over the primary motor cortex (M1) hot spot. (**c**) Magnetic resonance T1-weighted image (MRI) of one participant with targeted stimulation spot in the right lobule VIIIA at the cerebellar surface using radiological convention. (**d**) View from above. The black coil symbolizes the 25 mm branding-iron-coil positioned over the left PMd. White arrow indicates TMS current flow in anterior-to-posterior direction. Grey coil symbolized 70 mm coil positioned over M1 and slightly overlapping with PMd coil. White arrow indicates TMS current flow in posterior-to-anterior direction. (**e**) View from behind. The grey coil symbolizes the 70 mm coil positioned over the cerebellum (lobus VIIIA) with handle pointing cranially.

For cerebellar stimulation, we chose lobus VIIIA, as it was reachable via TMS (Fig. 2c), has been used as a target in other TMS studies^54^ and is important for the execution of motor tasks and learning processes^55^. All stimulation sites are shown in Fig. 2a.

### Transcranial magnetic stimulation

#### Single- and multi-pulse TMS

TMS pulses were generated by two Magstim 200^2^ and one Magstim 200 magnetic stimulator (Magstim Company, Whitland, Dyfed, UK). Left M1 was stimulated by a 70 mm figure-of-eight-shaped coil and left PMd by a 25 mm, branding-iron-style, figure-of-eight-shaped coil (‘‘baby coil”; Magstim Company, Whitland, Dyfed, UK) (Fig. 2b, d)^52,53^. The right cerebellum was stimulated by a 70 mm figure-of-eight-shaped coil^23^, positioned tangentially to the scalp with the handle directed upwards (Fig. 2b, e). We did not opt for a double-cone coil used to stimulate the cerebellum in some studies^56–59^, since five probands in a pilot study did not tolerate the stimulation due to pain and discomfort, and a figure-of-eight-shaped coil was frequently used in previous CBI studies^18,20,23,54,60–62^.

MEPs were generated by a supra-threshold intensity of 120% RMT and evoked an MEP of about 1 mV. Post-intervention MEPs were registered at the same stimulator output. RMT was identified as the intensity required to produce 5 out of 10 MEPs with an amplitude between 50 and 100 µV at a resting FDI for the 70 mm coil. AMT was identified as the intensity required to produce 5 out of 10 MEPs at > 150 µV at an activated FDI with 10% of maximum voluntary contraction using a Martin-Balloon-Vigorimeter (KLS Martin, Tuttlingen, Germany) for both the 70 mm and 25 mm coils. PMd-M1 interaction was probed with an ISI of 6 ms and an intensity of the conditioning pulse of 90% AMT^28,63^ and cerebello-M1 interaction at an ISI of 5 ms and an intensity of the conditioning pulse of 90% RMT^20,23,60,61^. Cerebello-PMd-M1 triple-pulse was administered with a PMd-M1 ISI of 6 ms and cerebello-M1 ISIs of 5, 7–10 and 12 ms using the same intensities for the conditioning pulse as stated above. Regarding the dual- and triple-pulse unconditioned MEPs, the test pulse intensity was adjusted to produce an amplitude of 1 mV pre and post plasticity induction.

#### Induction of plasticity

To induce plasticity, we used procedures previously described to be effective in inducing plasticity in the cerebellum. In experiment 1, 1 Hz rTMS^50^, PAS^17^, cTBS^44,51^, and anodal tDCS^64–66^ was used. 1 Hz rTMS was administered with 90% RMT for 20 min using a Magstim Rapid magnetic stimulator (Magstim Company, Whitland, Dyfed, UK)^50^.

PAS was performed for 30 min with an ISI of 6 ms between cerebellar and M1 TMS using an intensity of 110% RMT over the cerebellum and test pulse intensity over M1 as previously established^17^. The intertrial interval was 5 s.

CTBS was performed at 50 Hz for 40 s. at 80% AMT using a MagVenture MagOption (MagVenture, Lucernemarken, Denmark)^44,51^.

Anodal tDCS was administered with the anode placed 3 cm from the inion towards the right mastoid and the cathode on the right mandibula^64^, a montage frequently used in the past^65^ and shown to generate an efficient electric-field in simulations^67^. Stimulation was performed with a DC-Stimulator plus (neuroCare, Munich, Germany) for 20 min with an intensity of 1 mA (current density: 0.11 mA/cm^2^, total current of 2.2 mA/cm^2^^66^.

In experiment 2, 1 Hz rTMS and PAS were performed as in experiment 1. Additionally, a rTMS sham condition was performed using a sham coil. Sham PAS was administered for 30 min as established before^17^ with alternating ISIs of 2 and 10 ms between cerebellar and M1 stimulation using intensities as for real PAS.

### Data analysis and statistical analysis

Peak-to-peak amplitudes were measured for each trial. Conditioned MEPs were expressed as a percentage of unconditioned MEPs. For statistical analysis, multifactorial analysis of variance with repeated measures (ANOVA) using the factors INTERVENTION, TIME and ISI were performed. Greenhouse–Geisser correction was used to correct for non-sphericity. Post hoc student t-tests were performed using Bonferroni–Holm-correction if ANOVA resulted in a significant F value with *p* ≤ 0.05 for a main effect or interaction. In addition to separate analyses of experiments 1 and 2, a combined analysis of the 38 participants (two participants took part in both experiments) who received 1 Hz rTMS and PAS in experiments 1 or 2 was carried out. Factors used for the multifactorial analysis of variance with repeated measures (ANOVA) were INTERVENTION (experiment 1: rTMS, PAS, cTBS, tDCS; experiment 2: rTMS, sham rTMS, PAS, sham PAS), TIME (pre and post intervention) and ISI (for cerebellar-PMd-M1 triple-pulse only with ISIs of 5 ms, 7 ms, 8 ms, 9 ms, 10 ms, and 12 ms). For a combined analysis of rTMS and PAS effect, analysis was carried out as described above with the factors INTERVENTION using only rTMS and PAS.

For both types of stimulation, the mean stimulation effect was used as the basis for a sample size estimation for a potential group comparison study in patients using G*power^68,69^. We used usual values for the avoidance of a type 1 or type 2 error with an alpha error of 0.05, a power (1-beta) of 0.8 and an allocation ratio of 1:1. For the comparison group, it was postulated that there was no stimulation effect and that the variance of the effects would be equal to the group investigated in this study. Data are given as mean ± standard error of mean.

### Ethical statement

The study was approved by the ethic committee of the University of Lübeck and in accordance with the 1964 Helsinki declaration and its later amendments or comparable ethical standards. Informed and written consent of all participants was obtained.

## Results

Preintervention baseline data did not differ (*p* > 0.1) between sessions in any of the paradigms in either experiment (unconditioned MEPs, PMd-M1, cerebello-M1, cerebello-PMd-M1).

### Experiment 1—Comparison of four cerebellar plasticity protocols

#### Effects on unconditioned MEPs and motor thresholds

##### Effects on unconditioned MEPs

**Figure 3:**
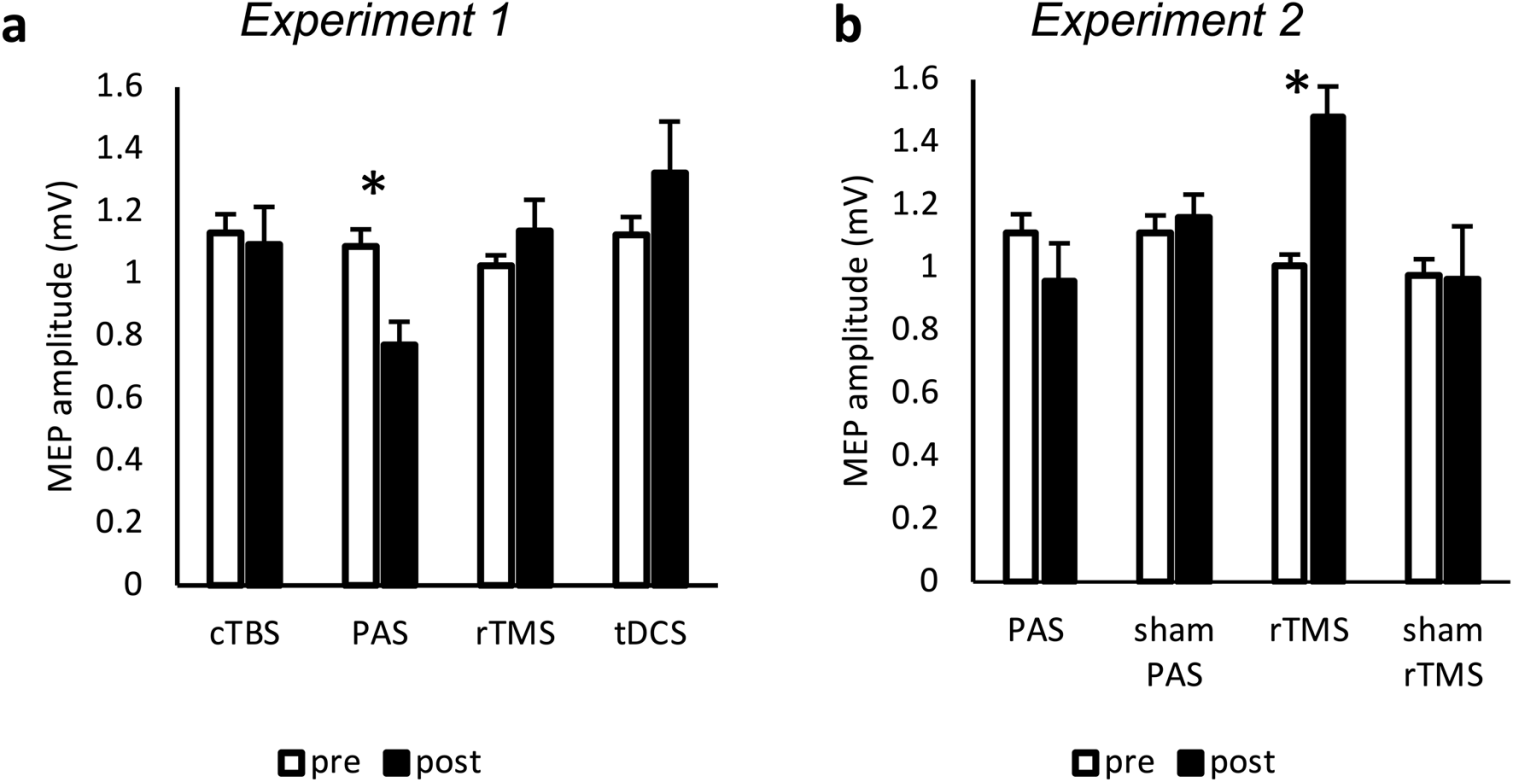
Analysis of MEPs (experiments 1 and 2). MEPs were measured peak-to-peak and are indicated in mV. Error bars indicate standard error of mean. Graphs are marked with * when comparison of values with student-t-test resulted in *p* value < 0.05 (Bonferroni–Holm-corrected). (**a**) Significant decrease of MEP amplitude after PAS intervention (*p* = 0.044). (**b**) Significant increase of MEP amplitude after rTMS intervention (*p* = 0.004). *MEP* motor evoked potential, *PAS* paired associated stimulation, *rTMS* repetitive transcranial magnetic stimulation, *cTBS* continuous theta-burst, *tDCS* transcranial direct current stimulation.

Analysis on unconditioned MEP amplitude using a multifactorial ANOVA showed a main effect for INTERVENTION (F(2.11,40.05) = 3.56, *p* = 0.036, $\upeta _{{\text{p}}}^{2}$ = 0.158) and an interaction of INTERVENTION and TIME (F(3,57) = 3.23, *p* = 0.029, $\upeta _{{\text{p}}}^{2}$ = 0.145). There was no main effect for TIME. Post hoc t-tests revealed a decrease of unconditioned MEP amplitudes following the PAS intervention (t = 2.62, *p* = 0.044) (Fig. 3a). TDCS, cTBS or rTMS had no significant effects on unconditioned MEP amplitudes (*p* > 0.1).

##### Effects on motor thresholds

Analysis of motor thresholds revealed an interaction between INTERVENTION and TIME for the AMT determined with the 25 mm coil (F(1.85,35.12) = 12.27, *p* < 0.001, $\upeta _{{\text{p}}}^{2}$ = 0.392) and the 70 mm coil (F(3,57) = 10.99, *p* < 0.001, $\upeta _{{\text{p}}}^{2}$ = 0.336) without a main effect for either TIME or INTERVENTION. Post hoc testing showed an increase after PAS intervention (25 mm coil: t =  − 6.13, *p* < 0.001, (44% ± 2.5% versus 47% ± 2.2% of maximum stimulator output (MSO)); 70 mm coil: t =  − 4.99, *p* < 0.001, 28% ± 1.4% versus 30% ± 1.3% MSO)) and a slight but significant decrease after rTMS intervention (only 70 mm coil: t = 3.17, *p* = 0.008, 28% ± 1.4% versus 27% ± 1.5% MSO). Analysis of RMT revealed a main effect of TIME (F(1,19) = 8.08, *p* = 0.010, $\upeta _{{\text{p}}}^{2}$ = 0.298) without interaction of INTERVENTION and TIME or a main effect of INTERVENTION.

#### Effects on conditioned MEP amplitudes

##### Effects on PMd-M1 interaction and on cerebello-M1 interaction

**Figure 4:**
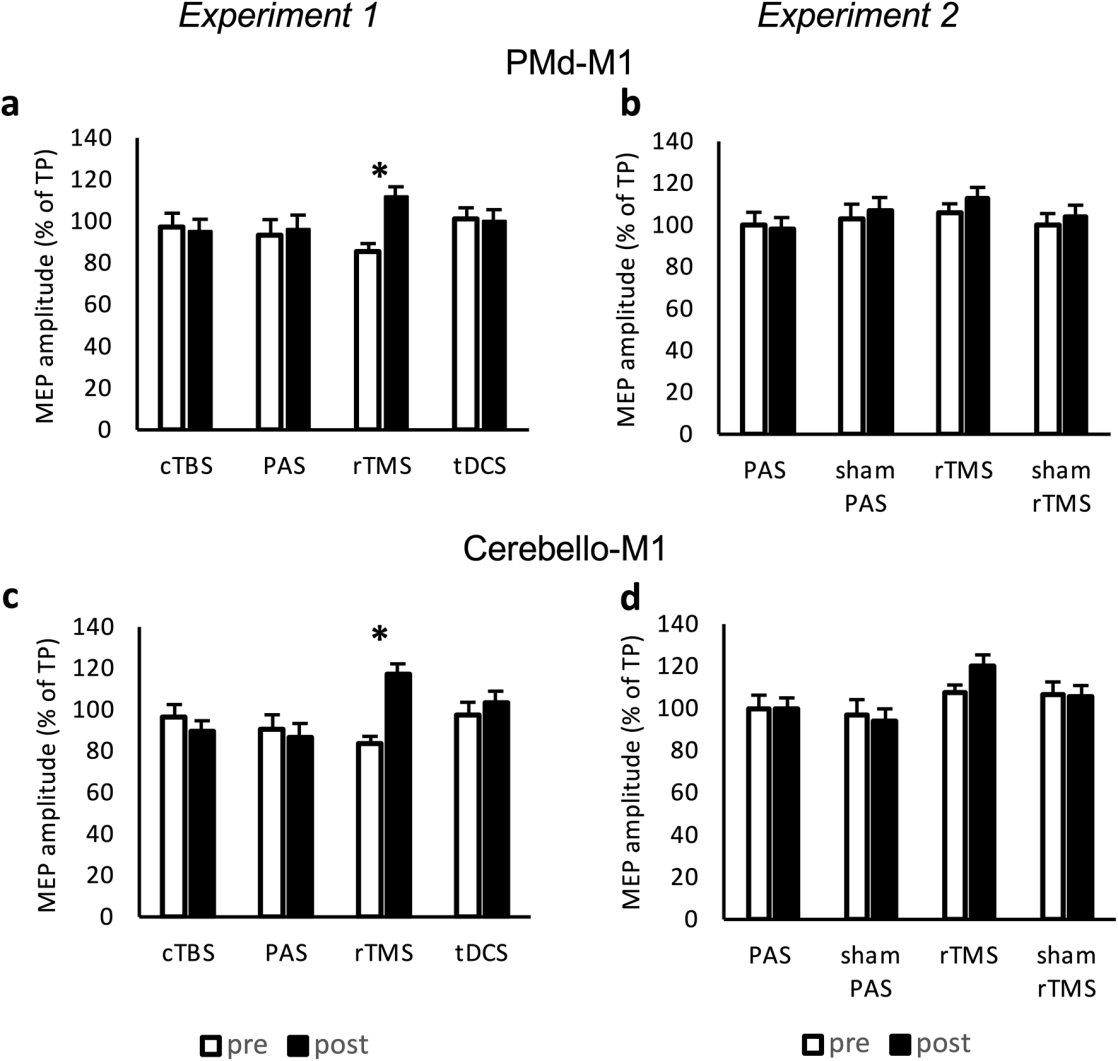
Analysis of dual-pulse TMS (experiments 1 and 2). MEP amplitudes were measured peak-to-peak and indicated in percentage to the MEP generated while only stimulating the primary motor cortex (M1) with a test pulse (TP). Conditioning pulses over PMd were administered before the TP over M1 within a dual-pulse PMd-M1 paradigm (**a** and **b**). Conditioning pulses were given over the cerebellum prior to TP M1 stimulation within the dual-pulse cerebello-M1 paradigm (c and d). Error bars indicate standard error of mean. Graphs are marked with * when comparison of values with student t-test resulted in *p* value < 0.05 (Bonferroni–Holm-corrected). (**a**) Significant increase of PMd-M1 interaction only after rTMS intervention (*p* = 0.004). (**b**) Increase of PMd-M1 interaction after rTMS intervention without significance (*p* = 0.250). (**c**) Significant increase of cerebello-M1 interaction after rTMS intervention (*p* < 0.001) (**d**) Increase of cerebello-M1 interaction after rTMS intervention with a trend to significance (*p* = 0.057). *MEP* motor evoked potential, *TP* test pulse over primary motor cortex, *PMd* dorsal premotor cortex, *M1* primary motor cortex, *PAS* paired associative stimulation, *rTMS* repetitive transcranial magnetic stimulation, *cTBS* continuous theta-burst, *tDCS* transcranial direct current stimulation, *ISI* interstimulus interval.

Analyzing conditioned MEPs in the PMd-M1 paradigm (relative MEP amplitudes at an ISI of 6 ms) ANOVA revealed an interaction between INTERVENTION and TIME (F(3,57) = 3.27, *p* = 0.028, $\upeta _{{\text{p}}}^{2}$ = 0.147) without a main effect of either INTERVENTION or TIME. The increase of relative MEP amplitudes following PMd conditioning after rTMS was significant in the post hoc test (t =  − 3.64, *p* = 0.004) (Fig. 4a).

Analysis of conditioned MEPs in the cerebello-M1 paradigm (relative MEP amplitudes at an ISI of 5 ms) revealed an interaction of INTERVENTION and TIME (F(3,57) = 6.54, *p* < 0.001, $\upeta _{{\text{p}}}^{2}$ = 0.256). The facilitatory effect after rTMS was significant in the post hoc test (t =  −4.42, *p* < 0.001) (Fig. 4c).

#### Effects on conditioned MEP amplitudes

##### Effects on cerebello-PMd-M1 interaction

**Figure 5:**
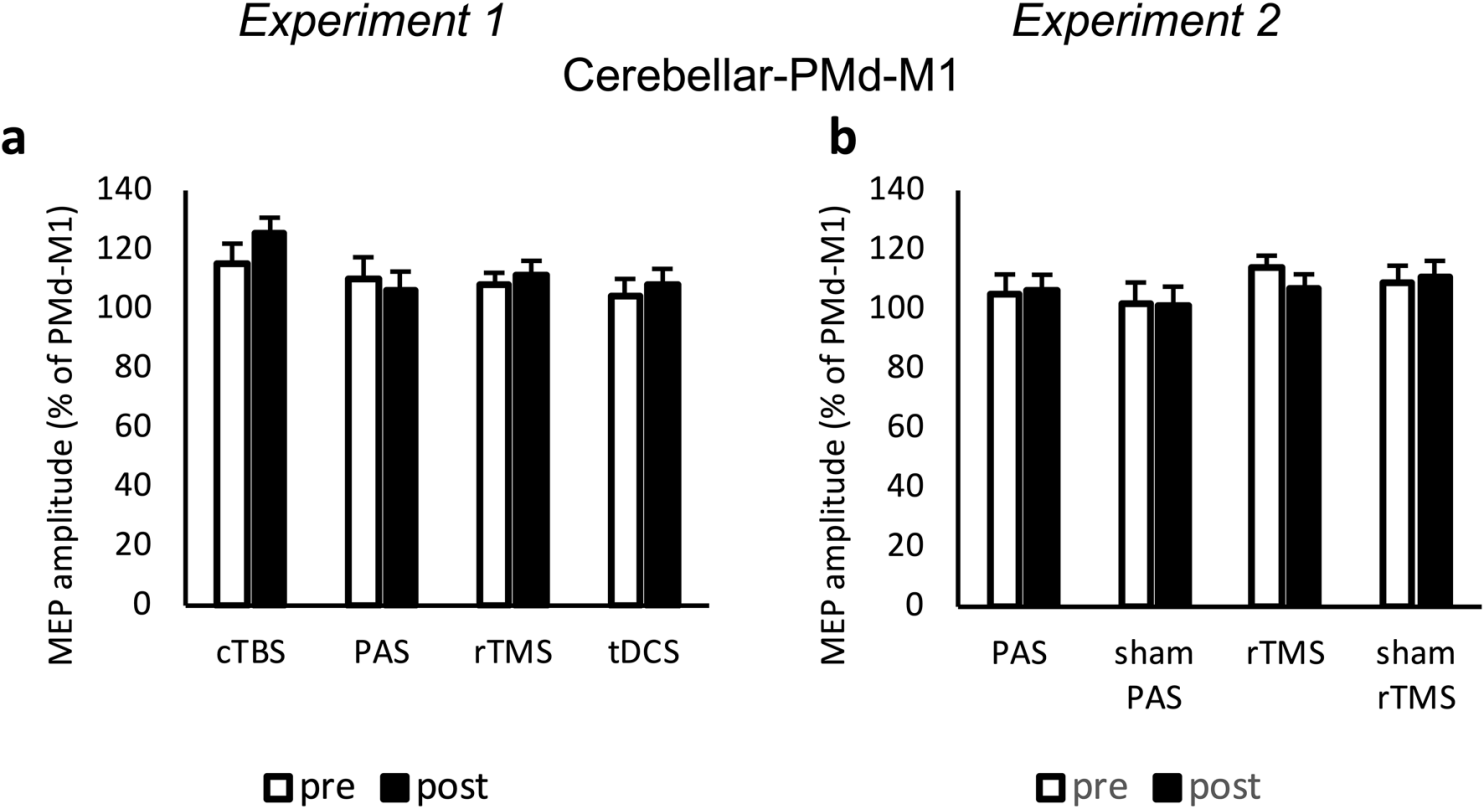
Analysis of triple-pulse TMS (experiments 1 and 2). MEP amplitudes were measured peak-to-peak and indicated in percentage to the MEP generated due to premotor cortex (PMd)-M1 interaction. Both PMd and cerebellar conditioning pulses were given in addition to M1 stimulation within the triple-pulse paradigm. Error bars indicate standard error of mean. (**a** and **b**) There was no significant change after any of the interventions. *MEP* motor evoked potential, *TP* test pulse over primary motor cortex, *PMd* dorsal premotor cortex, *M1* primary motor cortex, *PAS* paired associative stimulation, *rTMS* repetitive transcranial magnetic stimulation, *cTBS* continuous theta-burst, *tDCS* transcranial direct current stimulation, *ISI* interstimulus interval.

Analysis of conditioned MEP for cerebello-PMd-M1 triple-pulses (relative MEP amplitudes at ISIs between cerebellar and M1 stimulation of 5 ms, 7 ms, 8 ms, 9 ms, 10 ms, and 12 ms to PMd-M1 at 6 ms) did not show any main effects or interaction (Fig. 5a).

Taken together, in experiment 1, we found an inhibitory effect following PAS on unconditioned MEP amplitudes and a facilitatory effect of cerebellar 1 Hz rTMS on cerebello-M1 and PMd-M1 interactions.

### Experiment 2—Real versus sham rTMS and PAS

#### Effects on unconditioned MEPs and motor thresholds

##### Effects on unconditioned MEPs

Analysis of unconditioned MEP amplitudes showed a main effect for INTERVENTION (F(3,57) = 5.22, *p* = 0.003, $\upeta _{{\text{p}}}^{2}$ = 0.216), an interaction between INTERVENTION and TIME (F(3,57) = 6.36, *p* < 0.001, $\upeta _{{\text{p}}}^{2}$ = 0.251) but no main effect of TIME. Post hoc analysis revealed an increase of MEP amplitudes after rTMS (t =  − 4.31, *p* < 0.001) (Fig. 3b).

##### Effects on motor thresholds

In contrast to experiment 1, analysis of AMT values revealed a main effect of TIME for the 25 mm coil (F(1,19) = 9.84, *p* = 0.005, $\upeta _{{\text{p}}}^{2}$ = 0.341) but no interaction. There were no significant differences in AMT determined with the 70 mm coil.

For RMT, the main effect for INTERVENTION (F(3,57) = 3.62, *p* = 0.018, $\upeta _{{\text{p}}}^{2}$ = 0.160) and the interaction between INTERVENTION and TIME (F(3,57) = 3.73, *p* = 0.016, $\upeta _{{\text{p}}}^{2}$ = 0.164) were significant. Post hoc analysis for RMT showed a slight but significant increase of RMT after PAS (t = 3.39, *p* = 0.004, 39% ± 1.4% versus 40% ± 1.5%).

#### Effects on conditioned MEP amplitudes

##### Effects on PMd-M1 and on cerebello-M1 interactions

Analyzing conditioned MEPs for PMd-M1 and cerebello-M1 interaction revealed no main effects nor interaction (*p* > 0.1) (Fig. 4b, d).

##### Effects on cerebello-PMd-M1 interaction

Analysis of conditioned MEP for cerebello-PMd-M1 triple-pulse did not show any main effects or interaction (Fig. 5b).

Taken together, in experiment 2, we found a facilitatory effect following 1 Hz rTMS on unconditioned MEP amplitudes, but no significant effects on conditioned MEPs by rTMS or PAS. Sham protocols did not show any effect.

### Combined analysis of rTMS and PAS effects from experiments 1 and 2

#### Effects on unconditioned MEPs and motor thresholds

##### Effects on unconditioned MEPs

**Figure 6:**
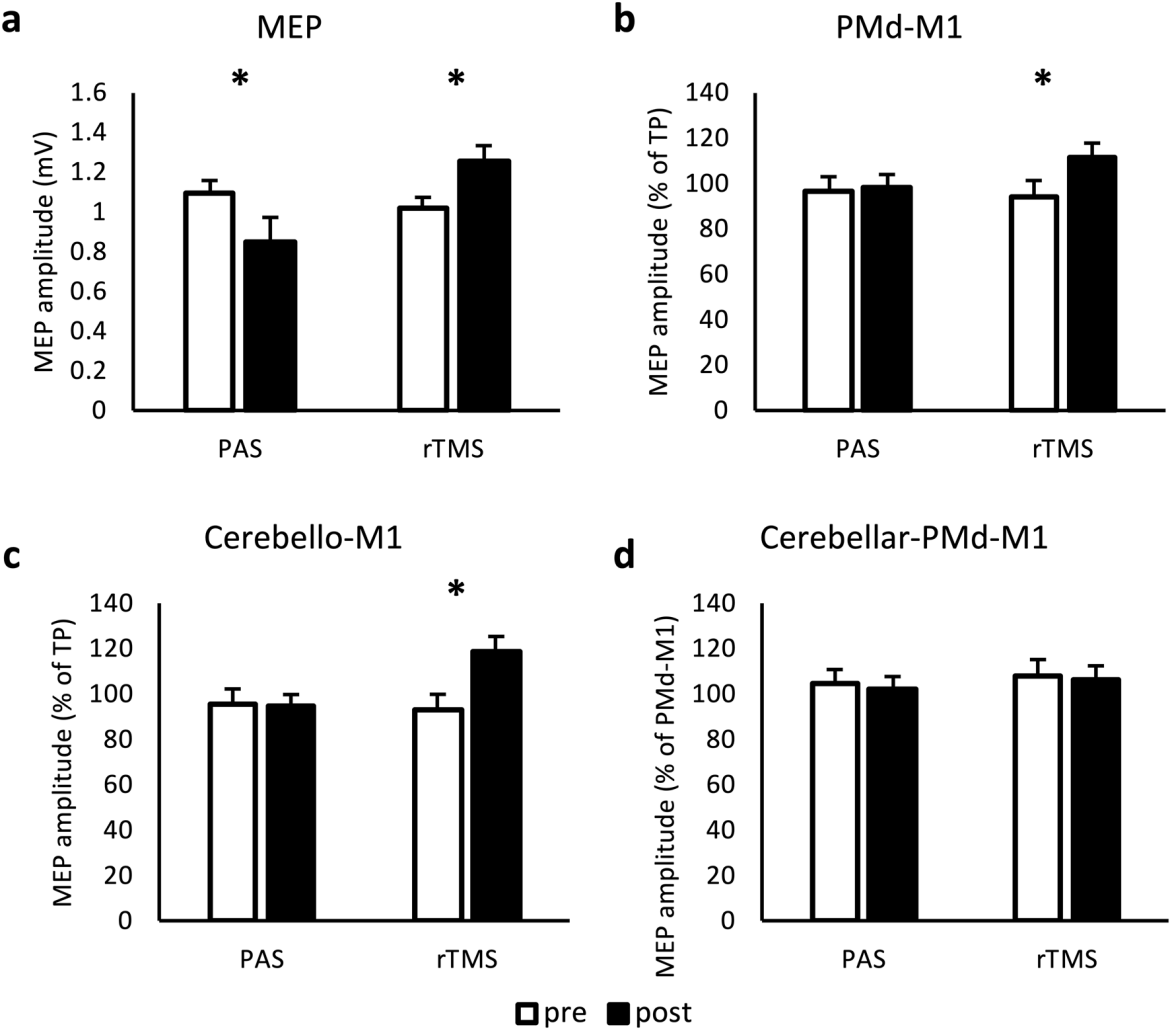
Analysis of MEPs and multi-pulse TMS in 38 participants (experiments 1 and 2). MEP amplitudes were measured peak-to-peak and are indicated in mV (**a**), in percentage to the MEP generated while only stimulating the primary motor cortex (M1) with a test pulse (TP) (**b** and **c**) or in percentage to the MEP generated due to premotor cortex (PMd)-M1 interaction (**d**). Conditioning pulses were administered before the TP on PMd (**b**), cerebellum (**c**) or both (**d**). Error bars indicate standard error of mean. Graphs are marked with * when comparison of values with student t-test resulted in *p* value < 0.05 (Bonferroni–Holm-corrected). (**a**) Significant decrease of MEP amplitude after PAS intervention (*p* = 0.005) and significant increase of MEP amplitude after rTMS intervention (*p* = 0.002). (**b**) Significant increase of PMd-M1 interaction after rTMS intervention (*p* = 0.002). (**c**) Significant increase of cerebello-M1 interaction after rTMS intervention (*p* < 0.001) (**d**) No significant change. *MEP* motor evoked potential, *TP* test pulse over primary motor cortex, *PMd* dorsal premotor cortex, *M1* primary motor cortex, *PAS* paired associative stimulation, *rTMS* repetitive transcranial magnetic stimulation.

ANOVA of unconditioned MEPs showed a main effect for INTERVENTION (F(1,37) = 12.26, *p* = 0.001, $\upeta _{{\text{p}}}^{2}$ = 0.249) and an interaction between INTERVENTION and TIME (F(1,37) = 19.98, *p* < 0.001, $\upeta _{{\text{p}}}^{2}$ = 0.351). There was no main effect for TIME. Post hoc analysis revealed a decrease of MEP amplitude following PAS (t = 2.73, *p* = 0.031) and an increase after rTMS (t =  − 3.48, *p* = 0.003) (Fig. 6a).

##### Effects on motor thresholds

ANOVA of motor thresholds revealed a main effect of TIME for AMT determined with the 25 mm coil (F(1,37) = 17.24, *p* < 0.001, $\upeta _{{\text{p}}}^{2}$ = 0.381) and RMT (F(1,37) = 16.39, *p* < 0.001, $\upeta _{{\text{p}}}^{2}$ = 0.307). There was no main effect of INTERVENTION or interaction between INTERVENTION and TIME for AMT determined both with the 25 mm and 70 mm coil as well as for RMT. Also, there was no main effect for TIME for AMT determined with the 70 mm coil.

#### Effects on conditioned MEP amplitudes

##### Effects on PMd-M1 and cerebello-M1 interactions

ANOVA on conditioned MEP amplitudes for PMd-M1 interaction showed a main effect for TIME (F(1,37) = 8.25, *p* = 0.007, $\upeta _{{\text{p}}}^{2}$ = 0.182) and an interaction between INTERVENTION and TIME (F(1,37) = 5.47, *p* = 0.025, $\upeta _{{\text{p}}}^{2}$ = 0.129). Post hoc analysis showed facilitation of PMd-M1 interaction after rTMS (t =  −3.70, *p* = 0.002) (Fig. 6b).

Analysis of cerebello-M1 interaction revealed a main effect of TIME (F(1,37) = 5.95, *p* = 0.020, $\upeta _{{\text{p}}}^{2}$ = 0.154) and INTERVENTION (F(1,37) = 6.73, *p* = 0.014, $\upeta _{{\text{p}}}^{2}$ = 0.139) and an interaction between INTERVENTION and TIME (F(1,37) = 13.17, *p* < 0.001, $\upeta _{{\text{p}}}^{2}$ = 0.263). Post hoc test showed a significant facilitation after rTMS (t =  −4.32, *p* < 0.001) (Fig. 6c).

##### Effects on cerebello-PMd-M1 interaction

Analysis of conditioned MEP for cerebello-PMd-M1 triple-pulse did not show any main effects or interactions of factors (Fig. 6d).

Taken together, in the combined analysis of experiment 1 and 2, we found a facilitatory effect following 1 Hz rTMS on unconditioned MEP amplitudes and on cerebello-M1 and PMd-M1 interactions, as well as an inhibitory effect following PAS on unconditioned MEP amplitudes.

### Sample size calculation for future group comparisons studies

In experiments 1 and 2, a total of 38 subjects were stimulated over the cerebellum with 1 Hz rTMS and PAS. This number of cases allowed us to estimate the sample size for potential comparative group studies with patients based on the stimulation effects on the MEP amplitudes found here. For the rTMS intervention, the change in MEP amplitude was 0.29 ± 0.59 mV and for PAS − 0.24 ± 0.44 mV. For a potential comparison group, we assumed that there were no stimulation effects in this group resulting in an effect size of dz = 0.49 for rTMS and dz = 0.55 for PAS. Based on these data, the required group size would be 66 participants per group for an rTMS intervention and 54 participants per group for the PAS intervention.

## Discussion

The first main finding of the present study is that 1 Hz rTMS over the right cerebellar lobule VIIIA increases corticospinal excitability as reflected by MEP amplitudes, whereas cerebellar PAS using two TMS pulses, one over the target area VIIIA followed by the second over M1, decreases MEPs. The second finding is that 1 Hz rTMS of right cerebellar lobule VIIIA also facilitates PMd-M1 and cerebello-M1 interactions. Similar to previous studies, this was not the case for the cerebellar PAS protocol^17^. Neither cTBS nor tDCS were effective in inducing plasticity supporting the results of previous studies, which also did not find effects on cortical excitability by cTBS^70^ or anodal tDCS^33,71^. As a third finding, we show that our results became clear and robust only with a sample size of 38, whereas results were quite variable and equivocal when only 20 participants were studied. This finding has considerable consequences for future studies using either cerebellar rTMS or cerebellar PAS as a treatment in clinical studies.

In line with our results, previous studies also found an increase of corticospinal excitability measured by MEPs after low-frequency rTMS stimulation over the cerebellum^42,43,72^, as well as abolishment of cerebello-M1 inhibition^70^. While our study focused on PMd-M1 and cerebello-M1 interaction, other studies investigated cortico-cortical interactions after cerebellar rTMS and found decreased intracortical facilitation at an ISI of 10 ms^72^ and an increase of intracortical facilitation at an ISI of 15 ms^42^. Both studies did not find alteration of SICI.

Probably, the mechanism of action of 1 Hz rTMS over the cerebellum is induction of plasticity resulting in transiently reduced excitability of Purkinje cells^42^, which leads to a decreased inhibitory effect on the dentate nucleus and therefore an increased excitatory output from the dentate nucleus to M1, an effect similar to long-term potentiation^30^. Based on single neuron and local field potential studies in cats and monkeys, it has been shown that the excitatory output of the cerebellum from the dentate nucleus projects onto excitatory as well as inhibitory interneurons in M1^12,73,74^. Previous studies suggested that interneuron population in M1 can be activated specifically by TMS, resulting in intracortical inhibition or facilitation^75,76^. The finding that cerebellar rTMS leads to both an increase of corticospinal excitability (reflected by an increase of unconditioned MEP amplitudes) and facilitation of PMd-M1 interactions suggests a specific effect on certain intracortical interneurons located in M1 but inter-connected with intracortical neurons located in the PMd. The increase of cerebello-M1 excitability after cerebellar rTMS could result from disinhibition of cerebellar to motor cortex pathway but also from remote effect on M1 interneurons. The fact that the combination of conditioned stimulation sites (cerebellum and PMd) had no additional effect on M1 or PMd-M1 excitability might be explained by both conditioning effects being mediated by the same interneuron network that is already optimally stimulated, i.e. might be caused by a ceiling effect.

Cerebellar PAS as used here apparently causes long-term depression-like effects reflected by an inhibition of corticospinal excitability (inhibition of MEPs and thresholds). PAS did not affect CBI or PMd-M1 interactions. Previous work has likewise found a clear effect of cerebellar PAS on corticospinal excitability, but no effects on CBI and SICI^17,77^. These results can be interpreted such that cerebellar PAS as used in our study with coupling of (preceding) cerebellar and M1 stimulation predominantly acts where the induced action potentials collide, i.e. within M1.

Importantly, rTMS and PAS effects in the present study were consistently present, either as a significant finding or as trends near significance in experiments 1 and 2, where 20 subjects each were studied, but became robust and unequivocal only with a group size of 38.

The effects of NIBS on the cerebellum have not been studied as extensively as those on other brain regions. Reported effects of interventions are partly contradictory^30,49,78,79^. One possible explanation is related to the anatomy of the cerebellum with proximity to neck muscles and a relatively large distance from the scalp to cerebellar Purkinje cells that are presumably the most relevant target for NIBS. This makes painless supra-threshold stimulation of the cerebellum difficult. Also, the size of the cerebellum renders specific stimulation of certain parts problematic. The exact stimulation regions are not specified in many studies^50,72,77,80–83^. Also, certain measurements such as CBI cannot be elicited in every person, or are not tolerated, so that these participants had to be excluded in previous studies. CBI is also very sensitive towards higher test pulse amplitudes that can reduce CBI. Given these drawbacks, information on criteria for the exclusion or inclusion of participants is important.

We tried to overcome the problem of spatial inaccuracy by using neuronavigation allowing us to more accurately define our target region, i.e. area VIIIA on the right cerebellar hemisphere. Moreover, we used a test pulse intensity of 120% RMT resulting in approximately 1 mV MEPs amplitudes. To increase specificity of our findings, we used four different NIBS techniques in experiment 1 and additionally sham stimulation in experiment 2. To reduce variability, our sample size was relatively large.

There are some limitations of our study. A limitation when studying intra-hemispheric PMd-M1 interaction is the close proximity between PMd and M1. Due to the size of the TMS coils optimal positioning over both targets is challenging. To avoid an anterior shift of the PMd coil, we first identified the individual anatomical PMd position in each subject and targeted the PMd using a 25 mm coil. Given the position of the PMd coil we adapted the angulation of the TMS coil and the stimulation intensity for M1 stimulation if necessary to keep the PMd conditioning accurate. Targeting the PMd is reliable on the basis of structural MRI. Therefore, we opted for anatomical targeting and recorded both positions (PMd and M1, Fig. 2). Alternatively, brain regions of interest can also be targeted on the basis of individual functional activation during tasks. For instance, previous studies on intra-hemispheric connections between M1 and the parietal cortex targeted TMS stimulation sites on the basis of brain activation during motor imagery^84^.

Another limitation is that we focused on PMd-M1 circuitry but did not examine other nodes or pathways that are also relevant for the understanding of plasticity in cerebellar-cortical networks including, for instance, the ventral premotor cortex, the supplementary motor area, and the posterior parietal cortex. These should be included in future studies.

Prior to the rTMS intervention, MEPs were not modulated by PMd or cerebellar conditioning. Unconditioned MEP amplitudes increased following rTMS, albeit non-significantly, which might nonetheless have influenced rTMS effects on PMd-M1 or cerebello-M1 interaction, i.e. facilitation.

We used the data of the change in corticospinal excitability following rTMS and PAS to estimate the number of cases needed to confirm or refute a stimulation effect with sufficient statistical power in a between-group comparisons, e.g. patients vs. healthy controls. We assumed that the findings of the present study would differ, i.e. would not be present, in a patient group. Therefore, 66 participants per group would be needed for a cerebellar rTMS and 54 for a cerebellar PAS intervention. In our calculation, we assumed the main effect to be quite large compared to a presumed zero change of MEP amplitudes after the intervention in a patient group. The variance of the effect was based on the data of this study, which we consider to be reliable, since we have chosen a non-selected group of subjects and have optimally controlled the stimulation conditions by means of neuronavigation.

Since the calculation of the required sample size depends on the assumed effect, e. g. the difference between groups, as well as the variance of the main effect within a given group, any change of these values would also affect the calculated number of cases needed., i.e. the number of required cases would be considerably larger if group differences of main stimulation effects were smaller.

These data suggest that the heterogeneity of published data on cerebellar NIBS could, at least partly, be explained by small numbers in many previous studies. It thus appears plausible and advisable to generally study larger cohorts in neurophysiological studies using rTMS and PAS as possible treatment options.

In summary, cerebellar 1 Hz rTMS increases net corticospinal excitability and facilitatory interactions in cerebello-M1 and PMd-M1 pathways, whereas cerebellar PAS reduces corticospinal excitability. Most likely, rTMS effects are mediated by long-term potentiation-like mechanism in cerebello-thalamo-cortical pathways projecting onto intracortical interneurons inter-connected with other cortical areas, whereas PAS probably leads to long-term depression-like effect in cerebello-thalamo-cortical pathways connected with neural elements primarily influencing pyramidal corticospinal output.

## Data Availability

The datasets generated during and/or analyzed during the current study are available from the corresponding author on reasonable request.

## Abbreviations

AMT: Active motor threshold
ANOVA: Analysis of variance
cTBS: Continuous theta-burst stimulation
FDI: First dorsal interosseus muscles
ISI: Interstimulus interval
M1: Primary motor cortex
MEP: Motor evoked potential
MSO: Maximum stimulator output
NIBS: Non-invasive brain stimulation
PAS: Paired associated stimulation
PMd: Dorsal premotor cortex
RMT: Resting motor threshold
rTMS: Repetitive transcranial magnetic stimulation
SICI: Short-interval intracortical inhibition
tDCS: Transcranial direct current stimulation
TMS: Transcranial magnetic stimulation
